# Task-Specific Facilitation of Cognition by Anodal Transcranial Direct Current Stimulation of the Prefrontal Cortex

**DOI:** 10.1093/cercor/bhv094

**Published:** 2015-05-15

**Authors:** Paul A. Pope, Jonathan W. Brenton, R. Chris Miall

**Affiliations:** School of Psychology, University of Birmingham, Birmingham, UK

**Keywords:** arithmetic cognition, dorsolateral prefrontal cortex, transcranial direct current stimulation, working memory

## Abstract

We previously speculated that depression of cerebellar excitability using cathodal transcranial direct current stimulation (tDCS) might release extra cognitive resources via the disinhibition of activity in prefrontal cortex. The objective of the present study was to investigate whether anodal tDCS over the prefrontal cortex could similarly improve performance when cognitive demands are high. Sixty-three right-handed participants in 3 separate groups performed the Paced Auditory Serial Addition Task (PASAT) and the more difficult Paced Auditory Serial Subtraction Task (PASST), before and after 20 min of anodal, cathodal, or sham stimulation over the left dorsolateral prefrontal cortex (DLPFC). Performance was assessed in terms of the accuracy, latency, and variability of correct verbal responses. All behavioral measures significantly improved for the PASST after anodal DLPFC stimulation, but not the PASAT. There were smaller practice effects after cathodal and sham stimulation. Subjective ratings of attention and mental fatigue were unchanged by tDCS over time. We conclude that anodal stimulation over the left DLPFC can selectively improve performance on a difficult cognitive task involving arithmetic processing, verbal working memory, and attention. This result might be achieved by focally improving executive functions and/or cognitive capacity when tasks are difficult, rather than by improving levels of arousal/alertness.

## Introduction

It is well established that the prefrontal cortex supports a wide variety of working memory (WM) functions, including the temporary storage and manipulation of visual and verbal material (Baddeley 1986, 1992). This is evidenced by prefrontal cortex activity on PET and fMRI during information processing tasks involving language (Petersen et al. 1988, 1989; Gabrieli et al. 1998; Smith et al. 1998; Wager and Smith 2003) or arithmetic (Kazui et al. 2000; Menon et al. 2000; Rickard et al. 2000; Kawashima et al. 2004; Arsalidou and Taylor 2011), and by many clinical observations of verbal WM deficits in patients with localized lesions of the prefrontal cortex (Müller and Knight 2006; Barbey et al. 2013). Frontal lobe lesions can also lead to poorer overall performance on tests of general arithmetic (Fasotti et al. 1992; Lucchelli and De Renzi 1993). Complementing this, transcranial direct current stimulation (tDCS) of the prefrontal cortex in healthy participants can facilitate cognition and improve certain aspects of performance in various WM tasks (Fregni et al. 2005; Flöel et al. 2008; Zaehle et al. 2011; Berryhill and Jones 2012; reviewed in Brunoni and Vanderhasselt 2014), leading researchers to employ tDCS as a therapeutic tool for treating depression (Brunoni et al. 2013) and cognitive deficits in patients after stroke (Jo et al. 2009), Parkinson's disease (Boggio et al. 2006), and with potential benefit after cerebellar dysfunction (reviewed in Pope and Miall 2014).

In short, tDCS in neurologically normal and intact participants is an alternative approach to studying brain–behavior relationships in patients with lesions, since it has the capacity to systematically modify behavior by inducing changes in underlying brain function. It involves delivering a weak direct current (DC) through a pair of electrodes: 1 stimulation electrode is placed over the region of interest, and the other reference electrode is placed on the head or shoulder on the opposite side of the body. Intracerebral current flow between the 2 electrodes excites neurons in the region of interest, producing both neurophysiological and behavioral changes in the participant. Over motor cortex, anodal stimulation of 1 mA for 9–13 min generally has an excitatory effect and increases cortical excitability, whereas cathodal stimulation generally has an inhibitory effect and decreases cortical excitability (Nitsche et al. 2003, 2008). Polarity-specific effects on cognition have also been reported after stimulating frontal regions of cortex (Javadi and Walsh 2012; Zwissler et al. 2014), albeit attributed by others to increases (anodal) or decreases (cathodal) in neuronal signal-to-noise ratio (Miniussi, Harris and Ruzzoli 2013). However, the aftereffects of tDCS in motor and cognitive domains are not always polarity-specific (Jacobson et al. 2012; Wiethoff et al. 2014).

We (Pope and Miall 2012) have previously shown how cathodal tDCS applied over the right cerebellum can facilitate performance in an attentionally demanding and difficult cognitive task known as the Paced Auditory Serial Subtraction Task (PASST), but not in the Paced Auditory Serial Addition Task (PASAT [Gronwall 1977]), which is less difficult to perform. We speculated whether this result was achieved with the release of cognitive resources by dis-inhibition of the left prefrontal cortex, because output from the cerebellar cortex to this frontal region is governed by the inhibitory Purkinje cells (Purves et al. 2001). But, the effects of electrical stimulation over the prefrontal cortex on performance during tests of numerical cognition are only just beginning to emerge (Snowball et al. 2013) and are still poorly understood. In fact, the question of whether tDCS over the prefrontal cortex can facilitate performance when an information processing task is more or less difficult to perform is in itself important to answer from the perspective of understanding the interaction between neuromodulation and cognitive load. The dorsolateral prefrontal cortex (DLPFC) is a good target within the prefrontal cortex, because it is activated by the PASAT as revealed by PET (Lockwood et al. 2004) and fMRI (Hayter et al. 2007). Other active regions include bilateral portions of the frontal and parietal lobes, superior temporal gyrus, the anterior cingulate, and bilateral cerebellar sites: regions consistent with elements of the task that include auditory perception and language processing, speech production, WM, and attention. Brain regions activated by the PASST have not been mapped, but performing mental subtraction in an MR scanner reveals activity in the left DLPFC, premotor cortex, Broca's area, and bilateral inferior parietal cortex (Burbaud et al. 1999).

Both addition and subtraction versions of the task involve participants listening to a series of numbers, and they are required to “add” or “subtract” the number they hear to or from the number presented immediately before it and then vocalize the answer. Both versions are difficult to perform and impose a high cognitive load, but they are typically achievable after a short practice block. The tasks also share the same overt speech operations (with comparable motor demands) but require different cognitive strategies. In fact, participants generally perform the subtraction task more slowly than the addition task, and they rate the PASST more difficult to perform than the PASAT (Pope and Miall 2012). In school children, learning to perform subtraction is also more difficult than learning to perform addition. Subtracting one number from another has 2 order-specific interpretations to consider, unlike adding 2 numbers together (Fuson, 1984), and subtraction can be achieved using multiple problem-solving strategies (Geary et al. 1993), which further differentiates subtracting from adding.

In this study, participants were asked to perform the PASAT and the PASST in a counterbalanced order at an individualized difficulty level (determined during practice to avoid ceiling effects), “before” and “after” the application of anodal, cathodal, or sham tDCS for 20 min over the left DLPFC in 3 separate groups. A between-subjects design was preferred because the PASAT is susceptible to practice effects over repeated measures (Tombaugh 2006). A visual analog scale (VAS) for assessing attention and mental fatigue was also used to investigate whether effects of tDCS on performance were due to changes in arousal/alertness over time (Ferrucci et al. 2013). We hypothesized that cognitive performance would be improved in a way that complimented the results from our previous study (Pope and Miall 2012). In other words, we predict that anodal tDCS over the left DLPFC would selectively facilitate cognition and improve performance in participants performing the PASST.

## Methods

The PASAT and the PASST were performed inside a quiet cubicle. Participants wore a headset (Beyerdynamic DT234 Pro) to minimize distractions, listen to auditory stimuli and to permit the recording and measurement of voice response onset times via the unidirectional microphone, which was gated by the amplitude of participants' verbal responses. The presentation of auditory stimuli and the recording of verbal responses was controlled using the Presentation^®^ software (Version 14.2, www.neurobs.com, last accessed on 29/4/15) running on a laptop computer. At the end of each session, participants rated their level of attention and mental fatigue on a visual analog scale (VAS). At the end of the experiment, they rated how difficult each task was to perform on a scale of 1 (easy) to 10 (difficult) and were then debriefed about the nature of the study.

### Participants

Sixty-three right-handed students (as determined by the hand used for writing) at the University of Birmingham participated for credit toward a psychology course requirement or for pay and were arbitrarily allocated into 3 groups of equal size, receiving anodal (8M/13F, mean age: 22.0, SD: 5.0 years), cathodal (7M/14F, mean age: 22.0, SD: 2.3 years), or sham (3M/18F, mean age: 21.4, SD: 3.8 years) stimulation. Four outlying female participants (1 from anodal, 1 from cathodal, and 2 from sham) were removed from data analysis, because response accuracy before tDCS on one of the tasks was outside the normal range as determined using the Shapiro–Wilk test. All participants were blind as to the type of stimulation they received, were instructed the same way, and gave informed consent, and the investigation was approved by the University of Birmingham Ethics Committee.

### Tasks

As in our previous study, participants performed a computer version of the PASAT (Gronwall 1977) and the PASST (Pope and Miall 2012), with a practice session at the start that included 45 items as opposed to the 10 practice items in the traditional version. The extra items allowed more time to assess the pace at which participants could perform each task, setting an individual rate within a certain limit to avoid a test ceiling effect. The instructions for the PASAT required participants to “add” the number they just heard to the number they heard before it. The instructions for the PASST required participants to “subtract” the number they just heard from the number they heard before it. The sequences used in each task before and after the stimulation period were different, and the numbers in the sequences were in the range of 1–9. Details of each task can be found elsewhere (Pope 2015).

### Visual Analog Scale

Participants rated their level of attention and mental fatigue on a VAS before and after stimulation. The VAS score ranged from 0 (best attention; no fatigue) to 100 (worst attention; worst fatigue) and was used as a subjective measure to assess arousal or alertness (tiredness). The scale consisted of a horizontal line on a printed sheet, 100 mm in length, and anchored at each end by a statement. The participant marked on the line the point they felt best represented their level of attention and mental fatigue at that moment. The VAS score was calculated by measuring the distance in millimeters from the left end of the scale to the point that the participant marked.

### tDCS

Stimulation was applied through 2 square sponge electrodes (surface area = 25cm^2^) moistened with saline solution in an air-conditioned room. The stimulation electrode was placed over the left DLPFC, corresponding to the electrode position F3 on the 10–20 international EEG system (Geary, Frensch and Wiley 1993). The reference electrode was placed on the right deltoid muscle to avoid the confounding effect of positioning 2 electrodes with opposite polarities on the brain (Javadi and Walsh 2012). This electrode montage would also avoid stimulation of right frontal and parietal sites that are active during arithmetic reasoning (Menon et al. 2000). The onset and offset of all interventions involved the current being increased and decreased, respectively, in a ramp-like manner over 10 s. The intensity of stimulation was set at 2 mA and delivered for 20 min using a Magstim DC Stimulator Plus. This intensity has been employed before over DLPFC during cognitive tasks (Boggio et al. 2006) and is considered safe and well below the threshold for causing tissue damage (Niedermeyer and Lopes da Silva 2004). Sham stimulation was applied for 20 min as per the built-in study function of the Magstim Stimulator (i.e., brief 15-ms pulses of 110 uA every 550 ms). During the stimulation period, participants were told to rest and not use electronic devices; they were not allowed to talk or read during this period.

### Procedure

Participants first received practice on both tasks. This also served to determine the rate at which items could be presented during the experiment without incurring too many errors. This was achieved by increasing the presentation rate of practice items (reducing the inter-stimulus interval by 300 ms) after every block of 5 items, between the interval range of 4.2 and 1.8 s. The interval at which each participant first made 3 errors in a row was noted, and the stimulus presentation rate preceding this cutoff point was then used in the experimental tasks and was kept the same in Session 1 (before stimulation) and Session 2 (after stimulation). This rate was therefore selected individually for each participant and each task. After practice on 1 task, participants performed the corresponding experimental task. The order participants performed the PASAT, and PASST was counterbalanced to ensure that performance on one task was not influenced by performance on the other. Session 1 included 2 practice and 2 experimental tasks, and the VAS and lasted ∼20 min with a short break in between tasks (∼30 s). Immediately after the stimulation period, participants performed Session 2, which included the 2 experimental tasks without practice and the VAS and lasted ∼10 min. Each answer was written down by the experimenter for subsequent verification, and correct answers were checked against a printed score sheet. No score was given if a participant gave an incorrect answer or failed to respond.

### Statistical Analysis

Results were analyzed in terms of the amount of change (difference) in performance between Session 1 and Session 2, to determine whether the mean change in the outcome from pre- to post-tDCS differed between tasks and groups. Difference scores were computed for each participant by subtracting their pre-tDCS score from their post-tDCS score, separately for the accuracy (percent of correct responses), latency (mean response times), and variability (standard deviation of mean response times) of participants' correct verbal responses. A positive difference score indicates that the post-tDCS score was greater than the pre-tDCS score, and a negative difference score indicates that the post-tDCS score was less than the pre-tDCS score. Difference scores for each measure were analyzed using separate 2 × 3 mixed-model ANCOVAs with Task (addition vs. subtraction) as a within-participant factor, and Group (Anodal vs. Cathodal vs. Sham) as a between-participant factor. Covariates (e.g., stimulus presentation rate for each task and gender) were added to the models to remove any effects they may have on the results. Subjectivity rating as a measure of task difficulty was not included as a covariate in the model because it was only collected in half the group. However, stimulus presentation rate was included instead as a measure of cognitive ability. Participants who performed the tasks more quickly at equivalent accuracy levels would presumably have greater WM and executive function skills than others who performed the tasks slowly. Additional analyzes were performed to determine whether the post-tDCS means, adjusted for the pre-tDCS means, differed between the 3 groups for each task separately using one-way ANCOVAs with post-tDCS scores as the dependent variable and pre-tDCS scores, cognitive ability, and gender as covariates. Two further participants from each group were also excluded from the analyses of response latency and variability because of technical issues or because they failed to complete both experiments before and after stimulation. Only correct answers were analyzed. Reasons for excluding individual trials included: incorrect, missed or inaudible/undetected responses, and double responses (i.e., a response preceded by lip movement/breath of air). The total amount of data excluded from data analysis was no more than 43% (37% incorrect and 6% other) for any 1 participant in any 1 session.

## Results

### Stimulus Presentation Rate

Given the unequal cognitive demands of performing the 2 tasks, participants' performed the subtraction task slower than the addition task (2.58 vs. 2.26 s) as confirmed with 2 × 3 (Task × Group) ANCOVA (with gender as a covariate) that found a significant main effect of Task, *F*_1,55_ = 16.27, *P* < 0.001. However, stimulus presentation rate did not differ significantly between the anodal, cathodal, or sham groups (2.40, 2.47, and 2.39 s, respectively, *F*_2,55_ = 0.34, *P* = 0.710). There was no Task by Group interaction for stimulus presentation rate, *F*_2,55_ = 0.11, *P* = 0.898. There was no possible session effect, as presentation rate was fixed across both sessions.

### Subjectivity Rating (Task Difficulty)

To confirm that the subtraction task is more difficult to perform than the addition task (as observed in our previous study [Pope and Miall 2012]), ratings of task difficulty from 9 participants in each group (anodal, 3M/6F, cathodal, 2M/7F sham, 2M/7F) were compared with ANCOVA (with stimulus presentation rate for each task and gender as covariates) between the 2 tasks. A main effect of Task, *F*_1,21_ = 5.04, *P* = 0.036, revealed that the subtraction task was rated significantly more difficult to perform than the addition task (6.93 vs. 5.15), suggesting an imbalance in cognitive load between tasks. There was no main effect of Group, *F*_2,21_ = 0.56, *P* = 0.577, or Task by Group interaction, *F*_2,21_ = 1.77, *P* = 0.195.

### Subjectivity Rating (Tiredness)

VAS scores for attention and mental fatigue from thirty-nine participants (anodal, 5M/8F, cathodal, 6M/8F sham, 2M/10F) were compared with ANCOVA (with stimulus presentation rate for each task and gender as covariates) between sessions to investigate whether tDCS influenced participants' subjective ratings of tiredness. A main effect of session, *F*_1,33_ = 1.33, *P* = 0.257, was not significant, suggesting that participants' subjective ratings of tiredness were not altered by tDCS between sessions (33.68 vs. 37.83). There was no main effect of Group, *F*_2,33_ = 0.34, *P* = 0.713, or Session by Group interaction, *F*_2,33_ = 0.42, *P* = 0.663.

### Difference Scores (Response Accuracy)

**Figure 1.**
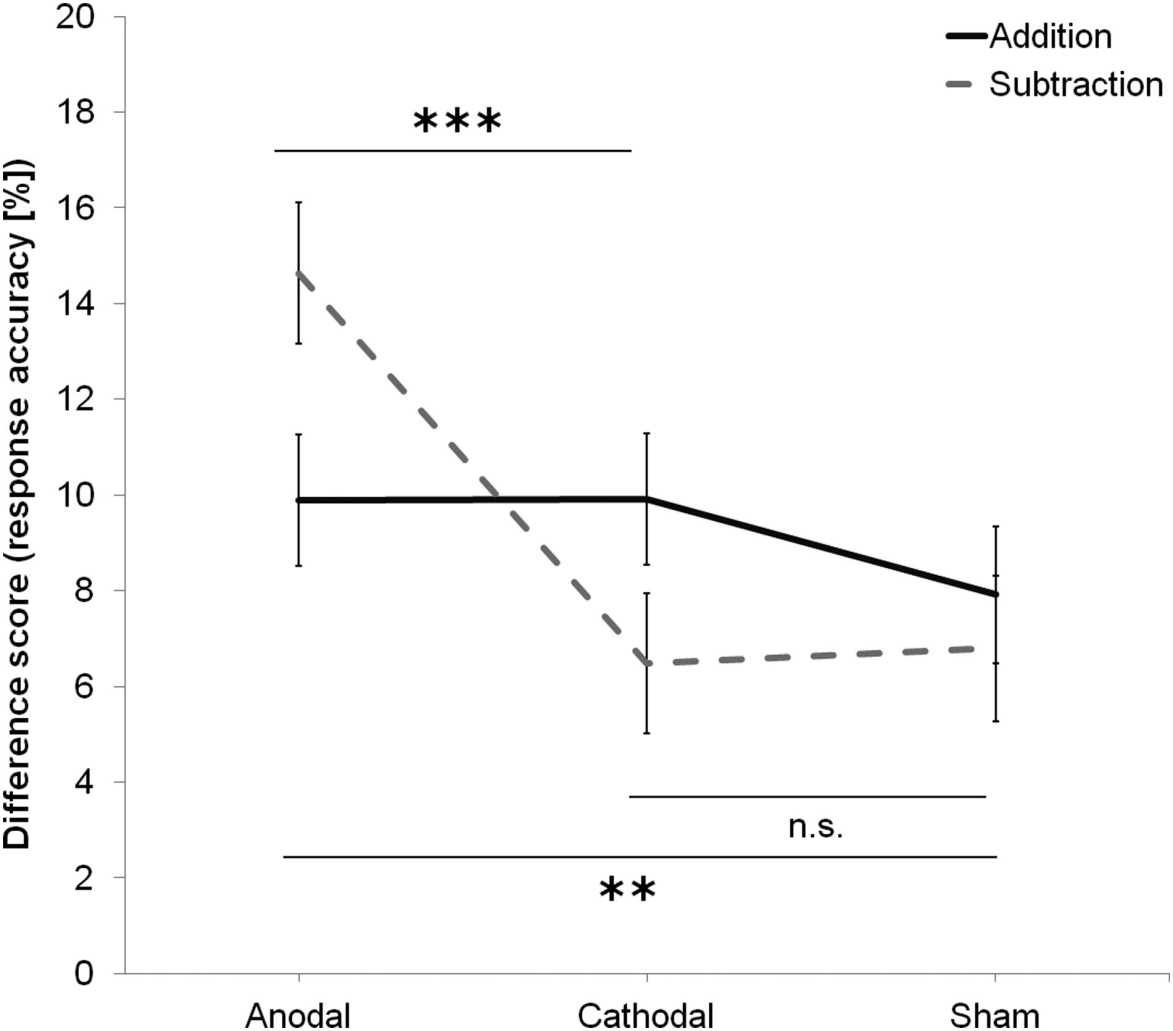
The increase in response accuracy (estimated marginal mean + 1 SEM) from Session 1 (before stimulation) to Session 2 (after stimulation), in the addition (PASAT) and subtraction (PASST) tasks, for each stimulation group. Participants achieved more correct answers in the subtraction task, after anodal stimulation, but not after cathodal or sham stimulation. Participants showed a nonselective improvement in the addition task in the second session, post-stimulation. Asterisks indicate significant two-tailed differences (*P* < 0.05) as revealed with pairwise comparisons (Bonferroni corrected).

The difference between sessions in the percent of participants' correct responses is summarized in Figure 1 for each group and task. A main effect of Group, *F*_2,53_ = 6.00, *P* = 0.004, revealed that the between-sessions increase in response accuracy differed significantly between groups. Pairwise comparisons (Bonferroni corrected) revealed significant differences between anodal and cathodal (12.26 vs. 8.20%; *P* = 0.026) and between anodal and sham (12.26 vs. 7.35%; *P* = 0.007), but not between cathodal and sham stimulation groups (8.20 vs. 7.35%; *P* = 1.00). Of interest was the Task by Group interaction that was also significant, *F*_2,53_ = 4.83, *P* = 0.012. Further pairwise comparisons (Bonferroni corrected) revealed that the between-sessions increase in response accuracy during the subtraction task differed significantly between anodal and cathodal (14.63 vs. 6.48%; *P* = 0.001), and between anodal and sham (14.63 vs. 6.79%; *P* = 0.002), but not between cathodal and sham stimulation groups (6.48 vs. 6.79%; *P* = 1.00), or between any group performing the addition task. There was no main effect of Task, *F*_1,53_ = 1.24, *P* = 0.271. The amount of change between sessions in response accuracy during the subtraction task following anodal stimulation is not due to differences in performance before stimulation as response accuracy pre-tDCS did not differ significantly between Task (73.69 vs. 74.10%; *F*_1,53_ = 0.05, *P* = 0.830) or Group (73.76 vs. 74.27 vs. 73.66%; *F*_1,53_ = 0.03, *P* = 0.972) as revealed by a separate two-way repeated-measures ANOVA.

### Pre-test/Post-test Comparisons (Response Accuracy)

For the PASST, ANCOVA revealed a main effect of Group, *F*_2,53_ = 11.87, *P* < 0.001. Pairwise comparisons (Bonferroni corrected) revealed that response accuracy post-tDCS differed significantly between anodal and cathodal (88.48 vs. 80.81%; *P* < 0.001) and between anodal and sham (88.48 vs. 80.92%; *P* < 0.001), but not between cathodal and sham stimulation groups (80.81 vs. 80.92%; *P* = 1.00), suggesting that anodal stimulation significantly increased response accuracy between sessions during the subtraction task. For the PASAT, ANCOVA revealed that there was no main effect of Group, *F*_2,53_ = 0.85, *P* = 0.435. Namely, response accuracy post-tDCS during the addition task was comparable between anodal, cathodal, or sham stimulation groups (83.60 vs. 83.72 vs. 81.52%).

### Difference Scores (Response Latency)

**Figure 2.**
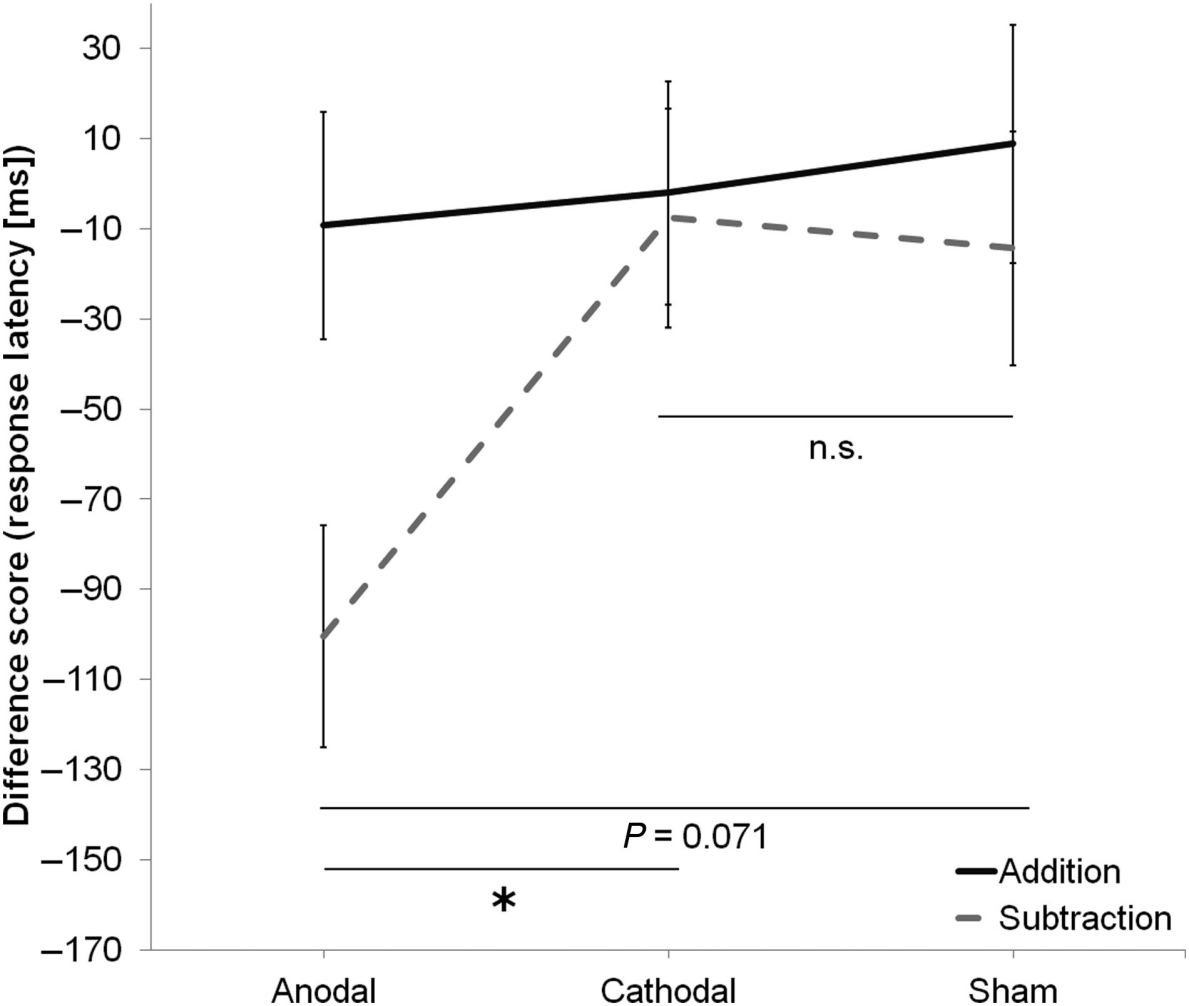
The reduction in response latency (estimated marginal mean + 1 SEM) from Session 1 (before stimulation) to Session 2 (after stimulation), in the addition (PASAT) and subtraction (PASST) tasks, for each stimulation group. Participants responded faster in the subtraction task, after anodal stimulation, but not after cathodal or sham stimulation. Participants showed a nonselective improvement in the addition task in the second session, post-stimulation. Asterisks indicate significant two-tailed differences (*P* < 0.05) as revealed with pairwise comparisons (Bonferroni corrected).

The difference between sessions in the timing of participants' correct responses is summarized in Figure 2 for each group and task. A main effect of Group was close to significance, *F*_2,47_ = 2.29, *P* = 0.11, such that there was a trend for the between-sessions reduction in response latency to differ between groups (−54.83 vs. −4.78 vs. −2.73 ms). The Task by Group interaction was not significant, *F*_2,47_ = 2.01, *P* = 0.146, but pairwise comparisons (Bonferroni corrected) revealed that the between-sessions reduction in response latency during the subtraction task differed significantly between anodal and cathodal (−100.40 vs. −7.56 ms; *P* = 0.029) and almost between anodal and sham (−100.40 vs. −14.33 ms; *P* = 0.071), but not between cathodal and sham stimulation groups (−7.56 vs. −14.33 ms; *P* = 1.00), or between any group performing the addition task. There was no main effect of Task, *F*_1,47_ = 1.00, *P* = 0.323. As before, a separate two-way repeated-measures ANOVA revealed that response latency pre-tDCS did not differ significantly between Task (1.30 vs. 1.41 s; *F*_1,53_ = 2.00, *P* = 0.164) or Group (1.33 vs. 1.36 vs. 1.37 s; *F*_1,53_ = 0.50, *P* = 0.611).

### Pre-test/Post-test Comparisons (Response Latency)

For the PASST, ANCOVA revealed a main effect of Group, *F*_2,47_ = 4.98, *P* < 0.011. Pairwise comparisons (Bonferroni corrected) revealed that response latency post-tDCS differed significantly between anodal and cathodal (1.30 vs. 1.40 s; *P* = 0.020) and between anodal and sham (1.30 vs. 1.41 s; *P* = 0.039), but not between cathodal and sham stimulation groups (1.40 vs. 1.41 s; *P* = 1.00), suggesting that anodal stimulation significantly reduced response times between sessions during the subtraction task. Again, there was no main effect of Group, *F*_2,47_ = 0.30, *P* = 0.744, for the PASAT. Namely, response times after tDCS were comparable between anodal, cathodal, or sham stimulation groups (1.27 vs. 1.32 vs. 1.31 s).

### Difference Scores (Response Latency Variability)

**Figure 3.**
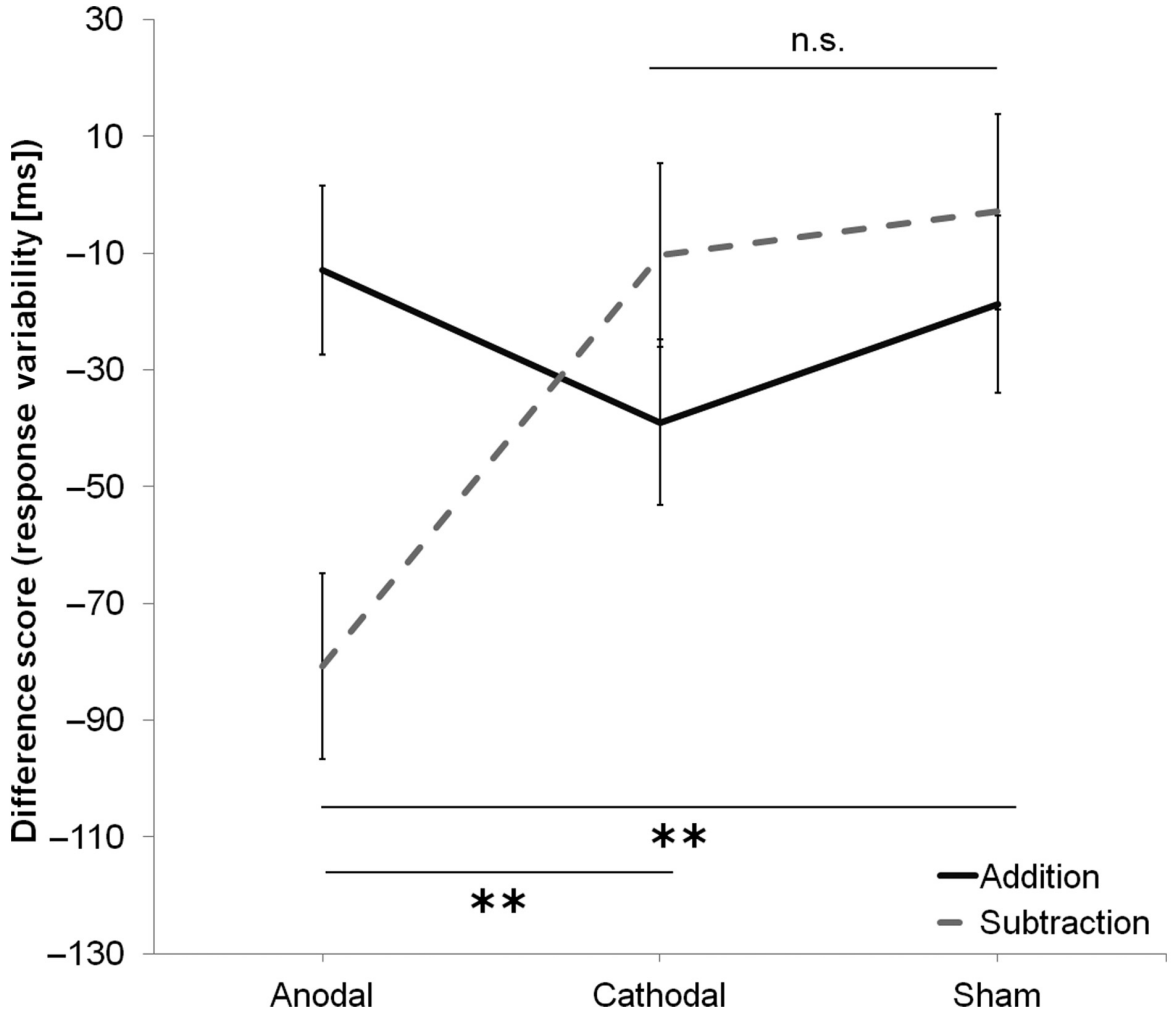
The decrease in response latency variability (estimated marginal mean SD + 1 SEM) from Session 1 (before stimulation) to Session 2 (after stimulation), in the addition (PASAT) and subtraction (PASST) tasks, for each stimulation group. Participants' response times were more consistent in the subtraction task, after anodal stimulation, but not after cathodal or sham stimulation. Participants showed a nonselective improvement in the addition task in the second session, post-stimulation. Asterisks indicate significant two-tailed differences (*P* < 0.05) as revealed with pairwise comparisons (Bonferroni corrected).

Figure 3 summarizes the difference between sessions in the consistency of participants' correct response latencies for each group and task. A main effect of Group was close to significance, *F*_2,47_ = 2.74, *P* = 0.075, such that there was a trend for the between-sessions decrease in response latency variability to differ between groups (−46.85 vs. −24.62 vs. −10.82 ms). Of interest, the Task by Group interaction was significant, *F*_2,47_ = 5.57, *P* = 0.007, and pairwise comparisons (Bonferroni corrected) revealed that the between-sessions decrease in response latency variability differed significantly between anodal and cathodal (−80.75 vs. −10.28 ms; *P* = 0.008) and between anodal and sham (−80.75 vs. −2.91 ms; *P* = 0.006), but not between cathodal and sham stimulation groups (−10.28 vs. −2.91 ms; *P* = 1.00) or between any group performing the addition task. There was no main effect of Task, *F*_1,47_ = 0.49, *P* = 0.488. And a separate two-way repeated-measures ANOVA revealed that the decrease between sessions in response latency variability during the subtraction task following anodal stimulation was not due to differences in performance before stimulation as response variability pre-tDCS did not differ significantly between Task (316.85 vs. 326.38 ms; *F*_1,47_ = 1.38, *P* = 0.246) and Group (325.48 vs. 324.28 vs. 315.07 ms; *F*_2,47_ = 0.16, *P* = 0.850).

### Pre-test/Post-test Comparisons (Response Latency Variability)

For the PASST, ANCOVA revealed a main effect of Group, *F*_2,47_ = 7.88, *P* < 0.001. Pairwise comparisons (Bonferroni corrected) revealed that post-tDCS response latency variability differed significantly between anodal and cathodal (249.16 vs. 318.73 ms; *P* = 0.003) and between anodal and sham (249.16 vs. 317.72 ms; *P* = 0.006), but not between cathodal and sham stimulation groups (318.73 vs. 317.72 ms; *P* = 1.00), suggesting that anodal stimulation significantly decreased response latency variability between sessions during the subtraction task. Once again, there was no main effect of Group, *F*_2,47_ = 0.99, *P* = 0.379, for the PASAT. Namely, response latency variability after tDCS was comparable between anodal, cathodal, and sham stimulation groups (301.24 vs. 281.88 vs. 296.20 ms).

## Discussion

The prefrontal and parietal cortices (among other regions) support cognitive processes necessary for performing arithmetic operations, as evidenced by neuroimaging studies (Burbaud et al. 1995, 1999; Menon et al. 2000; Zamarian et al. 2009). The prefrontal cortex is active during the learning of an arithmetic task and contributes to the control of WM and executive function skills necessary for supporting arithmetic processing, whereas parietal areas such as the angular gyrus are active during the retrieval of arithmetic facts and the solving of calculations per se (Zamarian et al. 2009). The results from the present study demonstrate that anodal stimulation over the left DLPFC can selectively improve performance in an attentionally demanding and difficult cognitive task involving mental subtraction known as the PASST, but not for the PASAT. Improvements after stimulation included an increase in the percent of correct responses (accuracy), and the responses were given faster and with less-variable latencies. The subtraction task was also rated more difficult to perform than the addition task. Participants' subjective rating of tiredness was not affected by the stimulation. The difficulty and increased attentional demands of subtraction tasks have recently been confirmed and exploited by others (Yasuda et al. 2014). We therefore suggest that transcranial anodal electrical stimulation of the DLPFC can affect performance when a cognitive task is attentionally demanding and especially difficult to perform, and perhaps when it engages specific WM operations. These results and ideas are discussed below.

The PASAT and the PASST share similar speech production processes, but the cognitive operations required to perform subtraction versus addition are very different. But, by individualizing the stimulus presentation rates for each task, participants were able to perform the PASST at a comparable level of accuracy to the PASAT. Thus, baseline accuracy in the PASAT and PASST was comparable, and performance in both tasks improved after stimulation, reflecting increased practice. However, after anodal stimulation, participants were able to perform the subtraction task with greater accuracy, faster and with greater consistency than any of the 3 groups performing the easier addition task. This result cannot be explained by a shift in attention or mental fatigue after the stimulation period, since participants' rating of tiredness was unaltered by tDCS. It suggests instead that the off-line effects of DLPFC-tDCS on cognition are likely task- or load-dependent: mediated perhaps by focally improving executive functions and/or increasing global workspace demands (i.e., cognitive capacity). This view parallels that of on-line effects of tDCS, which are thought to be sensitive to the state of the network that is active during stimulation (Miniussi, Harris and Ruzzoli 2013).

### Prefrontal Cortex Activity and Task Difficulty

To help address the present finding that tDCS over the DLPFC can improve performance when a cognitive task is attentionally demanding and especially difficult to perform, we note that previous brain imaging studies have revealed how activity in the prefrontal cortex is positively correlated with increased WM load (Dehaene et al. 1999) and task difficulty (Menon et al. 2000). Although the load maintained in WM is similar in the PASAT and the PASST (i.e., 2 digits), the problem-solving strategies required to perform the 2 tasks are very different. Order effects are relevant in subtraction (4 minus 3 is not the same as 3 minus 4), whereas they are irrelevant in addition, which is one reason why subtraction is more difficult to perform than addition (Fuson, 1984). Activity in the DLPFC also correlates with task difficulty during arithmetic tasks. For example, Menon et al. (2000) used a factorial fMRI design to explore activity related to task difficulty in arithmetic processing by manipulating the number of operands (2 vs. 3 operand equations) and the rate of stimulus presentation (3 vs. 6 s), so mapping brain regions that are unique to numeric computation or to task difficulty. They found activity associated with increasing task difficulty in the prefrontal and parietal cortices and the recruitment of additional brain regions, including the caudate and cerebellum bilaterally. Other brain imaging studies reveal how activity in a network comprising the frontal and parietal cortices is positively correlated with measures of increasing task difficulty such as reasoning and problem-solving (Rypma et al. 1999). There is also some evidence to suggest that tDCS can differentially modulate verbal WM in older participants as a function of strategy (Berryhill and Jones 2012). Most relevant, others have also shown how stimulating the left DLPFC can enhance solution generation of difficult problems, but not for easy problems (Metuki, Sela and Lavidor 2012).

### Cortical Networks for Arithmetic Operations

It is perhaps surprising that stimulating the DLPFC did not influence performance on the PASAT, which is known to be a difficult task to perform and recruits (among other areas) the prefrontal lobe (Lockwood et al. 2004; Hayter et al. 2007). Our results do not dispute the role of the DLPFC in executing the PASAT but imply that the effects of stimulating this region may not lead to detectable changes in performance if there are enough cognitive resources available for carrying out the task correctly. But, it is more likely, based on clinical studies, that distinct arithmetic operations rely on dissociated cortical networks. For example, some patients with parietotemporal damage show a selective deficit in tests of addition and multiplication in the face of a relative sparing of performance in subtraction tasks (Dagenbach and McCloskey 1992; Lampl et al. 1994; Pesenti et al. 1994). Network models of numerical cognition also posit that there are different routes in the brain for solving arithmetic problems. A direct asemantic route involving rote retrieval of arithmetic facts is proposed for solving simple addition and multiplication from language regions in the frontal lobes, and an indirect semantic route involving the retrieval of magnitude or quantity code from parietal lobes for solving subtraction and complex addition (Dehaene and Cohen 1995; Dehaene 1997). Finally, prefrontal areas and the anterior cingulate provide a global workspace for the sequential ordering of events through the processing stages: holding intermediate results in WM and detecting errors (Dehaene and Cohen 1997). It could be argued that global workspace demands are greater in subtraction than in addition, since sequential order effects are important when subtracting numbers, but irrelevant when adding numbers.

### Anodal Versus Cathodal Stimulation

Only anodal stimulation of the left DLPFC was found to selectively improve task performance in the present study. Performance after cathodal stimulation was not worse than after sham. However, the idea that anodal stimulation leads to excitation and cathodal stimulation leads to inhibition (as is common in motor studies) has not been confirmed in cognitive studies (Jacobson et al. 2012). This may partly be due to the fact that cognitive and physiological studies generally employ different stimulation intensities, durations, and electrode montages, and there is some evidence from motor studies that high cathodal stimulation can be excitatory (Wiethoff et al. 2014). Even motor responses induced by tDCS over motor cortex are highly variable between individuals, and not always polarity-specific (Wiethoff et al. 2014). Cathodal stimulation may not affect the cognitive operations employed in the present study, as we observed no effect in either the PASAT or PASST. This result is consistent with other experiments in which only anodal (and not cathodal) stimulation of the DLPFC was observed to improve WM performance (Fregni et al. 2005). Another study also reports cognitive improvements using an intensity of 2-mA and not 1-mA anodal stimulation over the DLPFC (Boggio et al. 2006).

However, present findings are the result of a single-session experiment. No follow-up measures were collected to investigate the duration of the cognitive after effects. In the clinic, where performance is assessed across multiple sessions with the aim of inducing long-lasting changes in behavior and cognition, there could be more profound effects. In the lab, repeated sessions of bihemispheric tDCS of M1 leads to increased speed and better force synchrony during motor learning, even 1 month after cessation of stimulation (Waters-Metenier et al. 2014). Alternative experimental designs are required to further investigate the durability of tDCS on cognition. A better understanding of individual factors that determine the efficacy and the mechanism of action of tDCS at different intensities is also required.

## Conclusion

We suggest that anodal tDCS over the left DLPFC can selectively facilitate cognition and improve performance when an attentionally demanding information processing task involving arithmetic processing (i.e., mental subtraction) and verbal WM is performed. We speculate that this is achieved by focally improving executive functions and/or increasing global workspace demands, since subjective ratings of arousal/alertness in each task were unchanged by tDCS over time. This result might be explained by the local increase in the excitability of the DLPFC.

## Funding

This work was supported by the Wellcome Trust (Grant ref. WT087554). Funding to pay the Open Access publication charges for this article was provided by the Wellcome Trust.

## References

[BHV094C1] Arsalidou M Taylor MJ 2011 Is 2+2=4? Meta-analyses of brain areas needed for numbers and calculations. Neuroimage. 54:2382–2393. 20946958 10.1016/j.neuroimage.2010.10.009

[BHV094C2] Baddeley AD 1986 Working Memory. Oxford: Clarendon Press.

[BHV094C3] Baddeley AD 1992 Working memory. Science. 255:556–559. 1736359 10.1126/science.1736359

[BHV094C4] Barbey AK Koenigs M Grafman J 2013 Dorsolateral prefrontal contributions to human working memory. Cortex. 49:1195–1205. 22789779 10.1016/j.cortex.2012.05.022 PMC3495093

[BHV094C5] Berryhill ME Jones KT 2012 TDCS selectively improves working memory in older adults with more education. Neurosci Lett. 521:148–151. 22684095 10.1016/j.neulet.2012.05.074

[BHV094C6] Boggio PS Ferrucci R Rigonatti SP Covre P Nitsche M Pascual-Leone A Fregni F 2006 Effects of transcranial direct current stimulation on working memory in patients with Parkinson's disease. J Neurol Sci. 249:31–38. 16843494 10.1016/j.jns.2006.05.062

[BHV094C7] Brunoni AR Valiengo L Baccaro A Zanão TA de Oliveira JF Goulart A Boggio PS Lotufo PA Benseñor IM Fregni F 2013 The sertraline vs electrical current therapy for treating depression clinical study: results from a factorial, randomized, controlled trial. JAMA Psychiatry. 70:383–391. 23389323 10.1001/2013.jamapsychiatry.32

[BHV094C8] Brunoni AR Vanderhasselt MA 2014 Working memory improvement with non-invasive brain stimulation of the dorsolateral prefrontal cortex: a systematic review and meta-analysis. Brain Cogn. 86:1–9. 24514153 10.1016/j.bandc.2014.01.008

[BHV094C9] Burbaud P Camus O Guehl D Bioulac B Caille JM Allard MA 1999 Functional magnetic resonance imaging study of mental subtraction in human subjects. Neurosci Lett. 273:195–199. 10515192 10.1016/s0304-3940(99)00641-2

[BHV094C10] Burbaud P Degreze P Lafon P Franconi JM Bouligand B Bioulac B Caille JM Allard M 1995 Lateralization of prefrontal activation during internal mental calculation: a functional magnetic resonance imaging study. J Neurophysiol. 74:2194–2200. 8592209 10.1152/jn.1995.74.5.2194

[BHV094C11] Dagenbach D McCloskey M 1992 The organization of arithmetic facts in memory: evidence from a brain-damaged patient. Brain Cogn. 20:345–366. 1449763 10.1016/0278-2626(92)90026-i

[BHV094C12] Dehaene S 1997 The number sense: how the mind creates mathematics. New York: Oxford University Press.

[BHV094C13] Dehaene S Cohen L 1997 Cerebral pathways for calculation: double dissociation between rote verbal and quantitative knowledge of arithmetic. Cortex. 33:219–250. 9220256 10.1016/s0010-9452(08)70002-9

[BHV094C14] Dehaene S Cohen L 1995 Towards an anatomical and functional model of number processing Math. Cogn. 1:83–120.

[BHV094C15] Dehaene S Spelk E Pine P Stanesc R Tsivkin S 1999 Sources of mathematical thinking: Behavioral and brain-imaging evidence. Science. 284:970–974. 10320379 10.1126/science.284.5416.970

[BHV094C16] Fasotti L Eling PA Bremer JJ 1992 The internal representation of arithmetical word problem sentences: frontal and posterior injured patients compared. Brain Cogn. 20:245–263. 1280445 10.1016/0278-2626(92)90019-i

[BHV094C17] Ferrucci R Brunoni AR Parazzini M Vergari M Rossi E Fumagalli M Mameli F Rosa M Giannicola G Zago S 2013 Modulating human procedural learning by cerebellar transcranial direct current stimulation. Cerebellum. 12:485–492. 23328908 10.1007/s12311-012-0436-9

[BHV094C19] Flöel A Rösser N Michka O Knecht S Breitenstein C 2008 Noninvasive brain stimulation improves language learning. J Cogn Neurosci. 20:1415–1422. 18303984 10.1162/jocn.2008.20098

[BHV094C20] Fregni F Boggio PS Nitsche M Bermpohl F Antal A Feredoes E Marcolin MA Rigonatti SP Silva MT Paulus W 2005 Anodal transcranial direct current stimulation of prefrontal cortex enhances working memory. Exp Brain Res. 166:23–30. 15999258 10.1007/s00221-005-2334-6

[BHV094C21] Fuson KC 1984 More complexities in subtraction. J Res Math Ed. 15:214–225.

[BHV094C22] Gabrieli JD Poldrack RA Desmond JE 1998 The role of left prefrontal cortex in language and memory. Proc Natl Acad Sci. 95:906–913. 9448258 10.1073/pnas.95.3.906 PMC33815

[BHV094C23] Geary DC Frensch PA Wiley JG 1993 Simple and complex mental subtraction: strategy choice and speed-of-processing differences in younger and older adults. Psychol Aging. 8:242–256. 8323728 10.1037//0882-7974.8.2.242

[BHV094C24] Gronwall DM 1977 Paced auditory serial-addition task: a measure of recovery from concussion. Percept Mot Skills. 44:367–373. 866038 10.2466/pms.1977.44.2.367

[BHV094C25] Hayter AL Langdon DW Ramnani N 2007 Cerebellar contributions to working memory. Neuroimage. 36:943–954. 17468013 10.1016/j.neuroimage.2007.03.011

[BHV094C26] Jacobson L Koslowsky M Lavidor M 2012 TDCS polarity effects in motor and cognitive domains: a meta-analytical review. Exp Brain Res. 216:1–10. 21989847 10.1007/s00221-011-2891-9

[BHV094C27] Javadi AH Walsh V 2012 Transcranial direct current stimulation (tDCS) of the left dorsolateral prefrontal cortex modulates declarative memory. Brain Stimul. 12:231–241. 21840287 10.1016/j.brs.2011.06.007

[BHV094C28] Jo JM Kim YH Ko MH Ohn SH Joen B Lee KH 2009 Enhancing the working memory of stroke patients using tDCS. Am J Phys Med Rehabil. 88:404–409. 19620953 10.1097/PHM.0b013e3181a0e4cb

[BHV094C29] Kawashima R Taira M Okita K Inoue K Tajima N Yoshida H Sasaki T Sugiura M Watanabe J Fukuda H 2004 Functional MRI study of simple arithmetic—a comparison between children and adults. Cogn Brain Res. 18:225–231. 10.1016/j.cogbrainres.2003.10.009 14741309

[BHV094C30] Kazui H Kitagaki H Mori E 2000 Cortical activation during retrieval of arithmetical facts and actual calculation: a functional magnetic resonance imaging study. Psychiatry Clin Neurosci. 54:479–485. 10997866 10.1046/j.1440-1819.2000.00739.x

[BHV094C31] Lampl Y Eshel Y Gilad R Sarova-Pinhas I 1994 Selective acalculia with sparing of the subtraction process in a patient with left parietotemporal hemorrhage. Neurology. 44:1759–1761. 7936312 10.1212/wnl.44.9.1759

[BHV094C32] Lockwood AH Linn RT Szymanski H Coad ML Wack DS 2004 Mapping the neural systems that mediate the Paced Auditory Serial Addition Task (PASAT). J Int Neuropsych Soc. 10:26–34. 10.1017/S1355617704101045 14751004

[BHV094C33] Lucchelli F De Renzi E 1993 Primary dyscalculia after a medial frontal lesion of the left hemisphere. J Neurol Neurosurg Psychiatry. 56:304–307. 7681473 10.1136/jnnp.56.3.304 PMC1014868

[BHV094C34] Menon V Rivera SM White CD Glover GH Reiss AL 2000 Dissociating prefrontal and parietal cortex activation during arithmetic processing. Neuroimage. 12:357–365. 10988030 10.1006/nimg.2000.0613

[BHV094C35] Metuki N Sela T Lavidor M 2012 Enhancing cognitive control components of insight problems solving by anodal tDCS of the left dorsolateral prefrontal cortex. Brain Stimul. 5:110–115. 22483547 10.1016/j.brs.2012.03.002

[BHV094C36] Miniussi C Harris JA Ruzzoli M 2013 Modelling non-invasive brain stimulation in cognitive neuroscience. Neurosci Biobehav Rev. 37:1702–1712. 23827785 10.1016/j.neubiorev.2013.06.014

[BHV094C37] Müller NG Knight RT 2006 The functional neuroanatomy of working memory: contributions of human brain lesion studies. Neuroscience. 139:51–58. 16352402 10.1016/j.neuroscience.2005.09.018

[BHV094C38] Niedermeyer E Lopes da Silva FH (eds). 2004 Electroencephalography: Basic Principles Clinical Applications and Related Fields, 5th edn Philadelphia: Lippincott Williams & Wilkins.

[BHV094C39] Nitsche MA Cohen LG Wassermann EM Priori A Lang N Antal A Paulus W Hummel F Boggio PS Fregni F 2008 Transcranial direct current stimulation: state of the art. Brain Stimul. 1:206–223. 20633386 10.1016/j.brs.2008.06.004

[BHV094C40] Nitsche MA Nitsche MS Klein CC Tergau F Rothwell JC Paulus W 2003 Level of action of cathodal DC polarisation induced inhibition of the human motor cortex. Clin Neurophysiol. 114:600–604. 12686268 10.1016/s1388-2457(02)00412-1

[BHV094C41] Pesenti M Seron X van der Linden M 1994 Selective impairment as evidence for mental organization of arithmetical facts: BB a case of preserved subtraction. Cortex. 30:661–671. 7697989 10.1016/s0010-9452(13)80242-0

[BHV094C42] Petersen SE Fox PT Posner MI Mintun M Raichle ME 1988 Positron emission tomographic studies of the cortical anatomy of single-word processing. Nature. 331:585–589. 3277066 10.1038/331585a0

[BHV094C43] Petersen SE Fox PT Posner MI Mintun M Raichle ME 1989 Positron emission tomographic studies of the processing of single words. J Cog Neurosci. 1:153–170. 10.1162/jocn.1989.1.2.153 23968463

[BHV094C44] Pope PA 2015 Modulating Cognition Using Transcranial Direct Current Stimulation of the Cerebellum. J Vis Exp (96), e52302. 10.3791/52302 PMC4354643 25741744

[BHV094C45] Pope PA Miall RC 2014 Restoring cognitive functions using non-invasive brain stimulation techniques in patients with cerebellar disorders. Front Psychiatry. 5:33. 24765079 10.3389/fpsyt.2014.00033 PMC3980102

[BHV094C46] Pope PA Miall RC 2012 Task-specific facilitation of cognition by cathodal transcranial direct current stimulation of the cerebellum. Brain Stimul. 5:84–94. 22494832 10.1016/j.brs.2012.03.006 PMC3379560

[BHV094C47] Purves D Augustine GJ Fitzpatrick D Katz LC LaMantia A McNamara JO Williams SM editors. 2001 Sunderland neuroscience, 2nd ed MA: Sinauer Associates.

[BHV094C48] Rickard TC Romero SG Basso G Wharton C Flitman S Grafman J 2000 The calculating brain: an fMRI study. Neuropsychologia. 38:325–335. 10678698 10.1016/s0028-3932(99)00068-8

[BHV094C49] Rypma B Prabhakaran V Desmond JE Glover GH Gabrieli JDE 1999 Load-dependent roles of frontal brain regions in the maintenance of working memory. Neuroimage. 9:216–226. 9927550 10.1006/nimg.1998.0404

[BHV094C50] Smith EE Jonides J Marshuetz C Koeppe RA 1998 Components of verbal working memory: evidence from neuroimaging. Proc Natl Acad Sci. 95:876–882. 9448254 10.1073/pnas.95.3.876 PMC33811

[BHV094C51] Snowball A Tachtsidis I Popescu T Thompson J Delazer M Zamarian L Zhu T Cohen Kadoshemail R 2013 Long-term enhancement of brain function and cognition using cognitive training and brain stimulation. Current Biology. 23:987–992. 23684971 10.1016/j.cub.2013.04.045 PMC3675670

[BHV094C52] Tombaugh TN 2006 A comprehensive review of the Paced Auditory Serial Addition Test (PASAT). Arch Clin Neuropsychol. 1:53–76. 16290063 10.1016/j.acn.2005.07.006

[BHV094C53] Wager TD Smith EE 2003 Neuroimaging studies of working memory: a meta-analysis. Cogn Affect Behav Neurosci. 3:255–274. 15040547 10.3758/cabn.3.4.255

[BHV094C54] Waters-Metenier S Husain M Wiestler T Diedrichsen J 2014 Bihemispheric transcranial direct current stimulation enhances effector-independent representations of motor synergy and sequence learning. J Neurosci. 34:1037–1050. 24431461 10.1523/JNEUROSCI.2282-13.2014 PMC3891947

[BHV094C55] Wiethoff S Hamada M Rothwell JC 2014 Variability in response to transcranial direct current stimulation of the motor cortex. Brain Stimul. 7:468–475. 24630848 10.1016/j.brs.2014.02.003

[BHV094C56] Yasuda K Sato Y Iimura N Iwata H 2014 Allocation of attentional resources toward a secondary cognitive task leads to compromised ankle proprioceptive performance in healthy young adults. Rehab Res Practice. Article ID 170304, 7 pages. 10.1155/2014/170304 PMC3910264 24523966

[BHV094C57] Zaehle T Sandmann P Thorne JD Jäncke L Herrmann CS 2011 Transcranial direct current stimulation of the prefrontal cortex modulates working memory performance: combined behavioural and electrophysiological evidence. BMC Neurosci. 12:2. 21211016 10.1186/1471-2202-12-2 PMC3024225

[BHV094C58] Zwissler B Sperber C Aigeldinger S Schindler S Kissler J Plewnia C 2014 Shaping memory accuracy by left prefrontal transcranial direct current stimulation. J Neurosci. 34:4022–4026. 24623779 10.1523/JNEUROSCI.5407-13.2014 PMC3951698

[BHV094C59] Zamarian L Ischebeck A Delazer M 2009 Neuroscience of learning arithmetic – evidence from brain imaging studies. Neurosci Biobehav Rev. 33:909–925. 19428500 10.1016/j.neubiorev.2009.03.005

